# Overweight among Medical Students of a Medical College

**DOI:** 10.31729/jnma.8305

**Published:** 2024-02-29

**Authors:** Ashish Bhattarai, Saroj Shrestha, Subodh Chapagain, Priya Yadav, Biswash Ratna

**Affiliations:** 1 Nepalgunj Medical College and Teaching Hospital, Kohalpur, Banke, Nepal; 2 Vayodha Hospital, Balkhu, Kathmandu, Nepal

**Keywords:** *body mass index*, *obesity*, *overweight*, *prevalence*

## Abstract

**Introduction:**

Overweight and obesity are rapidly increasing worldwide, posing a significant global health challenge. Medical students are at a higher risk of developing obesity due to factors such as a sedentary lifestyle, inadequate physical activity, unhealthy eating patterns, elevated stress levels, and the extensive amount of information they need to learn. The aim of this study was to find out the prevalence of overweight among medical students of a medical college.

**Methods:**

A descriptive cross-sectional study was conducted among medical students of a medical college from 5 October 2022 to 10 November 2022 after obtaining ethical approval from the Institutional Review Committee. Height in meters and weight in kilograms of students were measured to calculate body mass index. A convenience sampling method was used. The point estimate at a 95% Confidence Interval was calculated.

**Results:**

Among 261 medical students, 43 (16.47%) (11.97-20.97, 95% Confidence Interval) were overweight. Among them, 32 (74.41%) males and 11 (25.58%) females were overweight respectively.

**Conclusions:**

The prevalence of overweight among medical students is lower than in other studies done in similar settings.

## INTRODUCTION

Obesity is a multifactorial disease involving an excessive amount of body fat.^1^ The prevalence of obesity has been steadily increasing globally among different age groups and young people.^2^ Overweight and obesity are associated with more deaths globally than underweight. The global prevalence of obesity has experienced a nearly threefold increase since 1975.^3^

The study utilizes the Asian-specific criterion for assessing overweight and obesity. Obesity can increase the threat of diseases like hypertension, diabetes mellitus, dyslipidaemia, orthopaedic complications, gallstones, breast cancer, and psychological disorders.^4,5^

The aim of the study was to find out the prevalence of overweight among the medical students of a medical college.

## METHODS

This descriptive cross-sectional study was conducted among medical students of Nepalgunj Medical College (NGMC), Kohalpur, Banke, Nepal from 5 October 2022 to 10 November 2022, and ethical approval was obtained from the Institutional Review Committee of the same institute (Reference number: 14/079-080). Students studying Bachelor of Medicine and Bachelor of Surgery (MBBS) during the study period in NGMC were included in the study. Informed consent was taken from all the study participants. Students who did not give consent and those who were not present at the time of the study were excluded. A convenience sampling method was used. The sample size was calculated by using the following formula:


$$\begin{array}{ll} \text{n} & ={\text{Z}}^{2}\times \frac{\text{p}\times \text{q}}{{\text{e}}^{\text{2}}}\\ & =1.96^{2}\times \frac{0.50\times 0.50}{0.05^{2}}\\ & =385 \end{array}$$

Where,

n = minimum required sample size
Z = 1.96 for 95% Confidence Interval (CI)
p = prevalence of overweight for maximum sample size, 50%
q = 1-p
e = margin of error, 5%

The minimum sample calculated size was 385. The total number of students from first year to the intern (MBBS) is finite i.e. 477. Now, correcting the sample size for finite population:


$$\begin{array}{ll} \text{n} & =\frac{\text{n}}{1+\frac{\text{n}-1}{\text{N}}}\\ \text{n} & =\frac{\text{385}}{1+\frac{\text{385}-1}{\text{477}}}\\ & =214 \end{array}$$

Where,

n' = adjusted sample size for a finite population
N = finite population
n = calculated sample size

The adjusted sample size was 214. However, 261 sample sizes were taken.

The height of the subjects was measured using a stadiometer barefoot and wearing light clothes and was recorded in centimetres. The weight of the subjects was measured using a digital weighing machine and was recorded in kilograms.

According to the protocol, no shoes heel together; heel buttocks shoulder and head touching the vertical surface of the wall. The formula, weight in kilograms divided by the square of height in meters was used to calculate the BMI and the unit is kg/m^2^. According to the World Health Organization (WHO), "Asian Criteria" for BMI cut-off point 23-24.99 is overweight, pre-obese ≥25-29.9 and obese ≥30. This "Asian Criteria" is used to analyse overweight and obesity in this study.^6^

Data was entered into Microsoft Excel 2016 and IBM SPSS Statistics version 25.0. The point estimate was calculated at a 95% CI.

## RESULTS

**Figure 1:**
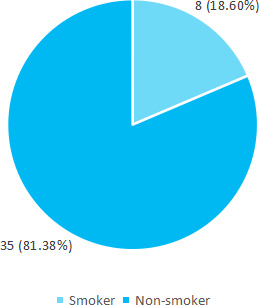
Smoking status of medical students (n = 43).

Among 261 medical students, 43 (16.47%) (11.97-20.97, 95% Confidence Interval) were overweight. The mean age was 22.34 ±2.04 years. Overweight males were 32 (74.41%) and females were 11 (25.58%) respectively. A total of 8 (18.60%) were smokers (Figure 1).

A total of 20 (46.51%) medical students consumed alcohol and 39 (90.69%) consumed a non-vegetarian diet.

## DISCUSSION

The prevalence of overweight was found to be 16.47% in medical students. In males, it was 74.41% and in females, it was 25.58%. This finding regarding prevalence is lower in comparison with the findings found in other studies done among medical students. A study done among 385 medical students in Kathmandu in 2021 showed the prevalence of overweight was 75 (19.48%).^7^ In another study done in Kerala India in 2019, the overall prevalence of overweight/obesity was found to be 30.6% in medical students. In males, it was 39.8% and in females, it was 25.5%.^8^ In another study done in Bengaluru, the prevalence of overweight was found to be 14.62% among medical students.^9^

Our study showed that the prevalence of overweight was found to be higher among male participants than their female counterparts. This could be due to the reason that males were proportionately higher than the females in the study sample. In our study, BMI was found to be higher in non-vegetarians. Previous studies conducted by multiple researchers support this finding.^10-12^

Since the study was conducted in a single institution, the findings of this study may not be generalized to the whole population. Our study design did not permit the measurement of association between variables. Hence, the authors would like to recommend conducting a similar study in multiple centres in the country with a large sample size.

## CONCLUSIONS

The prevalence of overweight among medical students was found to be lower than other studies done in similar settings. Further research, particularly involving multi-center studies with larger and more diverse samples, is recommended to gain a better understanding of the factors contributing to overweight among medical students and to draw more comprehensive conclusions.
